# Glances at the Hospitals

**Published:** 1901-12-28

**Authors:** 


### GLANCES AT THE HOSPITALS.

### NEWCASTLE-ON-TYNE HOSPITAL FOR SICK CHILDREN.

The income was £3,471, and the expenditure was £3,147. The total number of in-patients was 703, being 69 under that for the previous year. The average cost per in-patient was £4 3s., but this sum includes the cost of repairs and renewals, and if these be omitted the average cost would be reduced to £3 13s. per patient. The average stay of each patient in the hospital was 30 days. In the out-patients' department 6,873 patients were treated. The governors say that the work done in the hospital has been of a more serious nature than in any previous year, and they account for the average stay of patients in the hospital rising from 22 days to 30 days, by the fact that the demand for accommodation was so great that a large number of cases had been operated on and sent home the same day instead of being kept in the hospital. Of the 703 in-patients, 352 were discharged cured, 139 relieved, 84 not relieved, 57 died, and 71 remained under treatment. The medical and surgical tables are carefully compiled. We notice that excision of the hip joint was performed 7 times and that recovery followed in all. There were 9 amputations (2 of these being at the hip joint), and 8 recovered, 1 remaining under treatment. The average death rate in medical and surgical wards combined was 8·3 per cent. A "Shelter" for the treatment of consumptives has been erected here; and the medical staff state that the results have been encouraging. They add that, "prudence requires that, while the outdoor method is yet in a tentative stage of its development, care should be taken to avoid even the appearance of rashness in its use." We like caution in all things, but a system that has had twenty years' trial in Germany and about ten years in England, can hardly be described as in a tentative stage. Its value, or otherwise, ought to be fairly well-known by this time. The Swedish treatment for lupus has also been tried, but the absence of a powerful electric light has prevented any very decisive results at this institution. There ought to be sufficient rich men in Newcastle to present such a lamp to a hospital which evidently tries to keep abreast of modern treatment.

### MANCHESTER CLINICAL HOSPITAL.

The receipts amounted to £3,861, and the expenditure to £3,853, showing a small balance on the right side, and placing the institution in a better position than it was in the two previous years when large deficiencies occurred; but this result is more apparent than real, because a donation of £1,000 was placed to income instead of to investment. The governors add that "the financial position of the hospital is a source of grave anxiety to them, and that the amount received from subscriptions is steadily diminishing." A new departure in the history of the hospital has taken place by the appointment of a lady (Miss A. M. Roberts, M.B.) as house surgeon, and as the hospital is entirely for women and children we look upon the appointment as a very satisfactory one. The medical and surgical tables are drawn out to show the number of cases suffering from the various diseases, the number cured, the number died, and the number remaining under treatment. Ovariotomy was performed 8 times, and oophorectomy 15 times, all being successful. Chloroform seems to be the anæsthetic used in this hospital.

